# Ketamine and Dexmedetomidine Combination for the Management of the Pediatric Difficult Airway

**DOI:** 10.7759/cureus.50473

**Published:** 2023-12-13

**Authors:** Rodrigo Ferreira, Margarida Telo, Joana Figueiredo

**Affiliations:** 1 Anesthesiology, Hospital da Luz Lisboa, Lisbon, PRT

**Keywords:** pediatric difficult airway, airway, ketodex, dexmedetomidine, ketamine

## Abstract

The pediatric difficult airway is a challenge for the anesthesiologist. In this article, we describe a case where ketamine and dexmedetomidine were used to approach a difficult airway in a five-month-old patient with a palatal teratoma. These two drugs have complementary effects, because of which they can be used to maintain ventilation without compromising airway reflexes and are suitable for the management of pediatric difficult airways.

## Introduction

The pediatric difficult airway remains a challenge for the anesthesiologist and is an important contributor to both patient morbidity and mortality. Critical respiratory events are common in children, although the incidence of failed tracheal intubation remains low [1]. In pediatric patients with craniofacial abnormalities or tumors, the probability of a difficult airway is much higher, but data pertaining to its real incidence are sparse.

The best method to approach a pediatric difficult airway is still debatable. Some advise that the induction of general anesthesia may be as safe as sedation. Sedation practices vary widely, and one of the major difficulties is guaranteeing adequate intubation conditions with minimal airway reactivity while maintaining spontaneous respiration. When sedation is tried to approach a pediatric airway, there is a high incidence of conversion to general anesthesia for successful tracheal intubation [2].

We describe a case of a pediatric patient with a palatal tumor proposed for surgical excision. We focus our report on the management of the airway and the sedation used to approach this clinical scenario.

## Case presentation

A five-month-old boy with the diagnosis of a primary palatal teratoma was proposed for surgical excision. The patient weighed 7.8 kg and had no other relevant medical history. The American Society of Anesthesiologists (ASA) physical status classification of III was attributed to the presence of a difficult airway. The tumor was circumscribed to the hard palate and extruded from the mouth, leaving only a small free space on the left side of the mouth.

Face mask ventilation was impossible due to the size of the teratoma, and there was a risk of airway patency loss if a standard induction was used. Plan A was nasal fiberoptic intubation. Plan B was a videolaryngoscopy, and plan C was a tracheostomy. The airway management plan was established in conjunction with pediatric anesthesiologists, ENT surgeons, and pediatric surgeons. The team had predefined roles; a senior pediatric anesthesiologist was responsible for plans A and B, and the ENT team would do the tracheostomy if needed.

Since our plan A was nasal fiberoptic intubation, a sedation plan that allowed airway manipulation in an uncooperative pediatric patient was needed. Our major goal was to maintain spontaneous ventilation with minimal airway reactivity.

For sedation, a mixture of ketamine (1 mg/kg) and dexmedetomidine (1 mg/kg), diluted in a 10 cc syringe, was the chosen option. Afterward, an infusion of ketamine 0.2 to 2 mg/kg/h and dexmedetomidine 0.2 to 2 mg/kg/h in a 50 cc syringe with an infusion pump would be started.

Standard ASA monitors were applied to the patient, who already had IV access. Over 10 minutes, 8 mg of ketamine and 8 mg of dexmedetomidine were administered as a bolus. Immediately after, the infusion of ketamine at 1.5 mg/kg/h and dexmedetomidine at 1.5 mg/kg/h was started in an infusion pump. Topical anesthesia was applied using a nasal atomizer with lidocaine 0.5% in 4 mL in both nares. Glycopyrrolate 30 mg (4 mg/kg) and dexamethasone 1.5 mg (0.2 mg/kg) were administered intravenously with the goal of antisialagogue and anti-inflammatory properties, respectively (Table 1).

**Table 1:** Drugs used for airway management

Drugs used for airway management	
Sedation and analgesia	Ketamine 1 mg/kg + dexmedetomidine 1 mcg/kg IV bolus for over 10 minutes
	Ketamine 1.5 mg/kg/h + dexmedetomidine 1.5 mcg/kg/h IV perfusion
Topical anesthesia	Lidocaine 0.5% 8 mL (nasal atomizer)
Antisialagogue	Glycopyrrolate 4 mcg/kg IV
Corticosteroid	Dexamethasone 0.2 mg/kg IV

For oxygenation and monitoring of the expired CO_2_, an uncuffed 2.5 mm endotracheal tube was placed in the left nostril and connected to the anesthesia machine.

**Figure 1:**
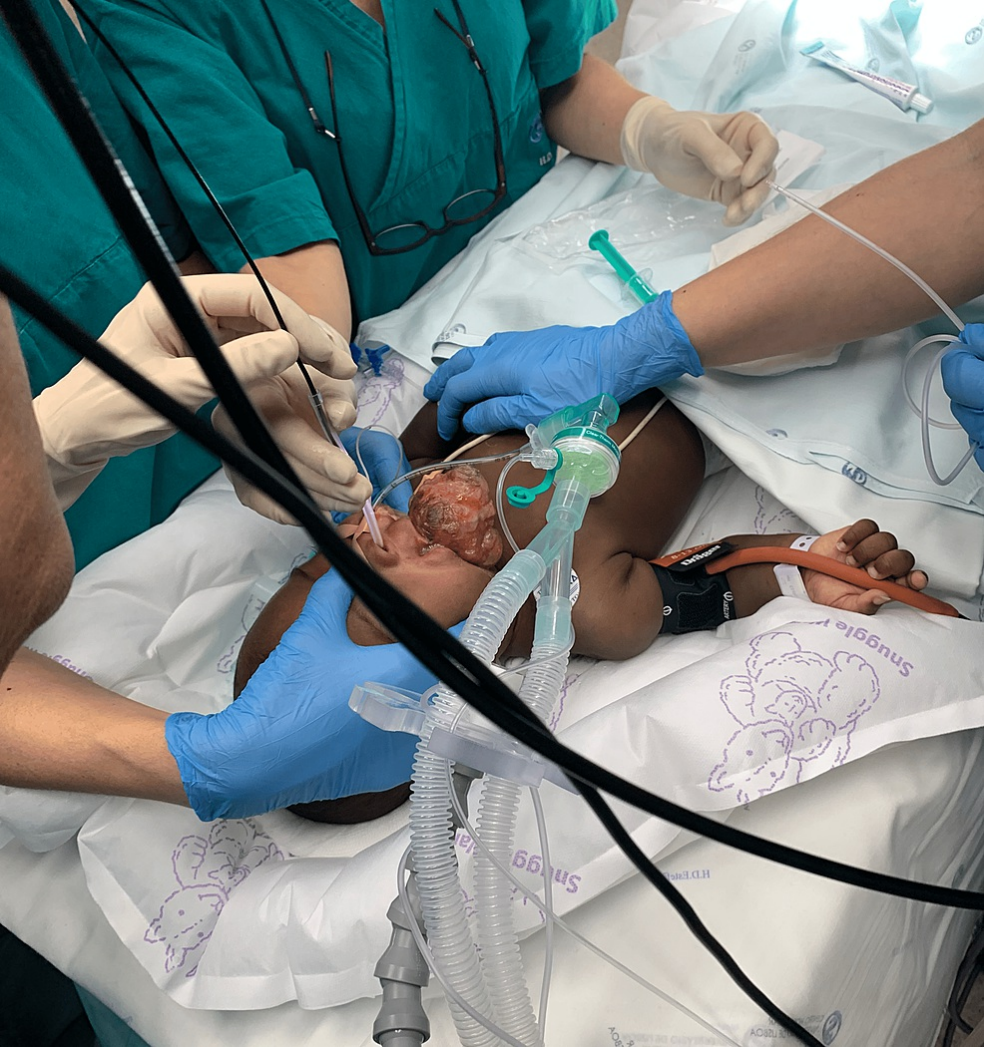
Nasal fiberoptic intubation

Nasal fiberoptic intubation was initiated (Figure 1), and the patient remained adequately sedated.

**Figure 2:**
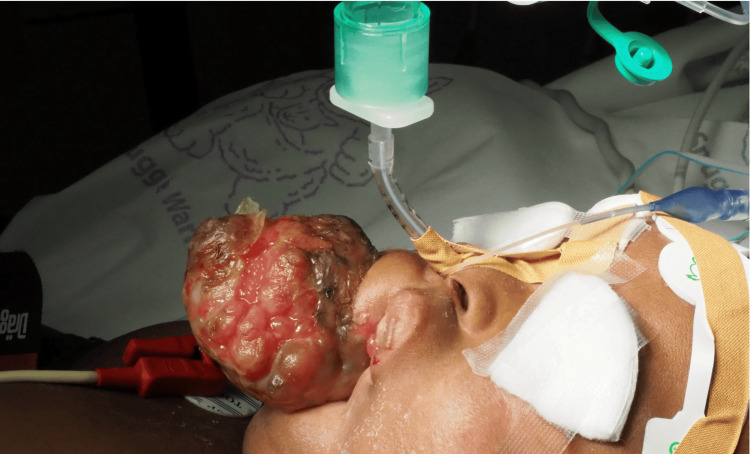
The patient after securing the airway and anesthesia induction

However, it was technically challenging. We were able to see the vocal cords but were unable to advance the endotracheal tube to the trachea. The small oral and nasal space in a five-month-old may have contributed to the inability to place the tube. The decision was made to change to plan B and use a videolaryngoscope from HugeMed® (Shenzhen HugeMed Medical Technical Development Co., Ltd., Shenzhen, China) with a pediatric blade. The videolaryngoscope was inserted through the small mouth opening and the nasotracheal intubation was successful after the first attempt. Induction of anesthesia was performed with fentanyl and propofol and the patient was connected to the anesthesia machine (Figure 2).

During the intubation attempts, the sedation was adequate, achieving state two on the Pediatric Sedation State Scale (PSSS). No episodes of apnea, desaturation, or airway reactivity were documented. Capnography was present with proper waveform during all the airway procedures, and vital signs remained stable.

**Figure 3:**
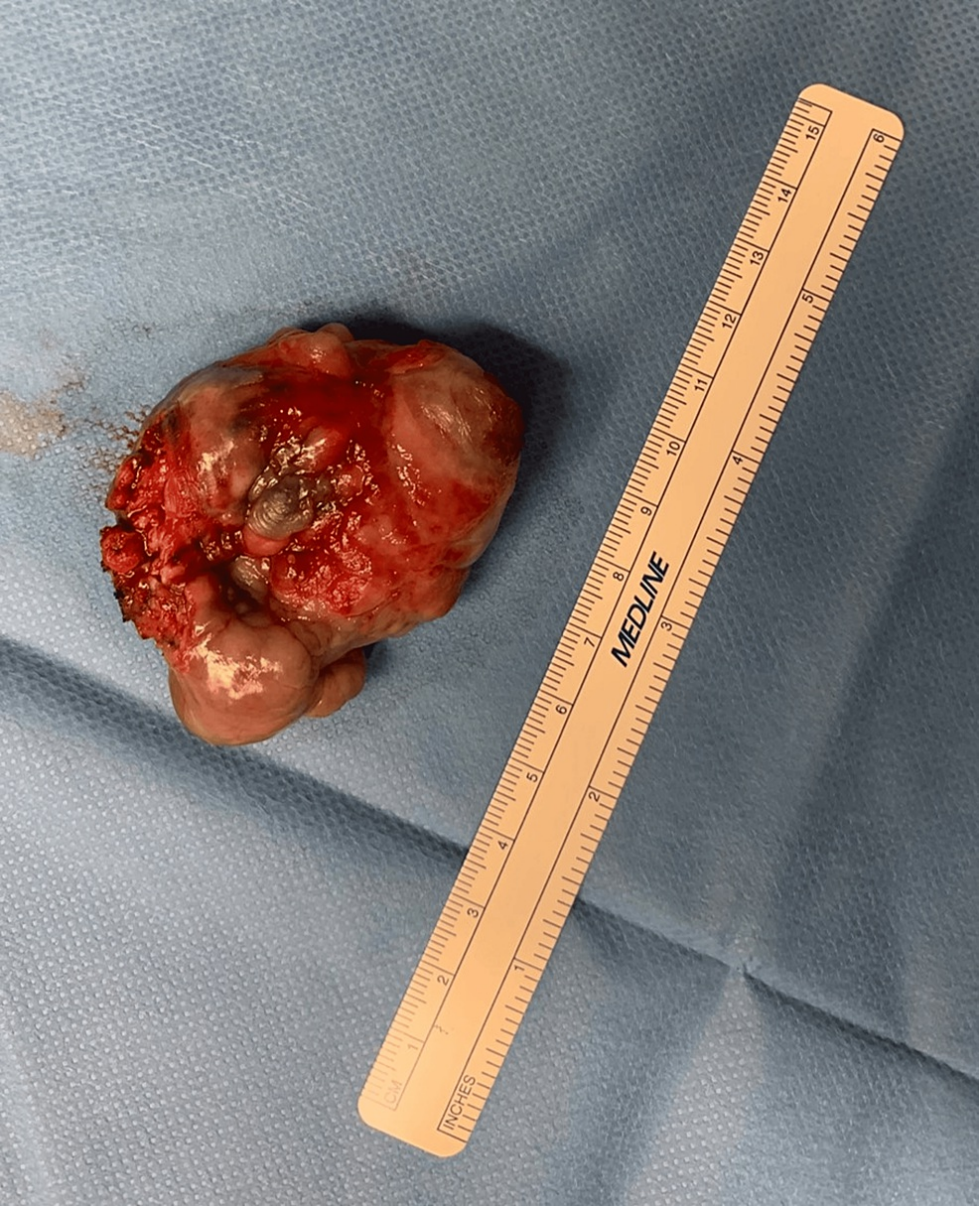
Palatal teratoma after excision

**Figure 4:**
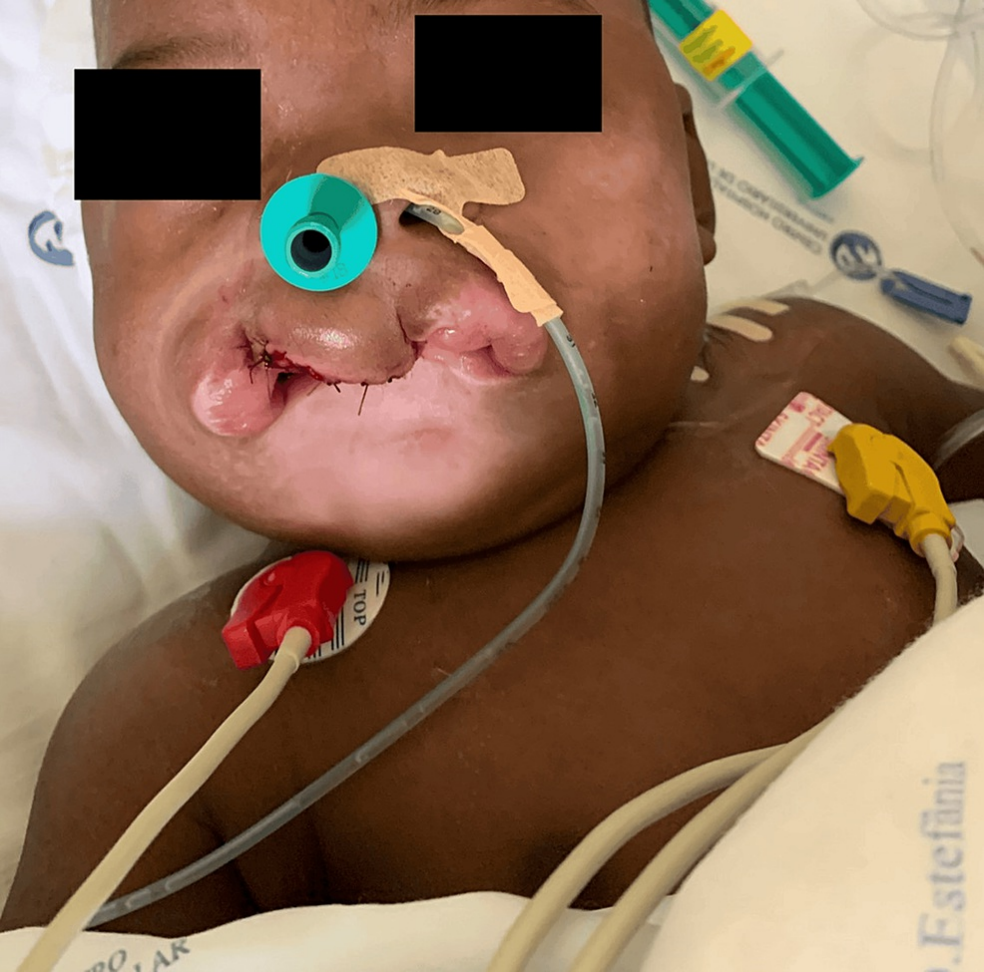
Patient after extubation at the post-anesthesia care unit (PACU)

When surgery was completed, the patient was extubated and transferred to our post-anesthesia care unit (PACU) (Figures 3, 4).

## Discussion

The pediatric difficult airway can be categorized into two scenarios: the unanticipated and the anticipated difficult airway. Both have common management pathways. However, the anticipated difficult airway can be carefully prepared. The establishment of an airway plan is associated with better outcomes [3].

The establishment of plans A, B, and C in a multidisciplinary team (ENT surgeons, pediatric surgeons, and pediatric anesthesiologists) allowed us to smoothly follow a plan when faced with a challenging airway. Given the inability of children to cooperate, we managed to find a sedation technique that made it possible to perform nasal fiberoptic intubation and videolaryngoscopy on a spontaneously breathing patient.

Ketamine and dexmedetomidine sedation are used in many contexts for sedation in children, for instance, while performing upper gastrointestinal endoscopy and administering regional anesthesia [4-6]. The use of this combination provides excellent hemodynamic stability and maintenance of spontaneous ventilatory drive [7].

Ketamine is a competitive inhibitor at the N-methyl-D-aspartate (NMDA) receptor and has side effects that cause tachycardia, hypertension, hypersalivation, and an increased incidence of agitation [4]. Dexmedetomidine is a selective alpha-2-adrenoceptor agonist associated with bradycardia and hypotension and has an antisialagogue effect [8]. The combination of these two drugs allows the side effects to balance off each other, with low effects on cardiovascular and ventilatory function, making them a perfect combination [7]. Both drugs have analgesic properties that are useful, and dexmedetomidine also reduces ketamine-induced agitation [9].

Another effect observed in this case was the ability to provide adequate levels of sedation and analgesia without compromising spontaneous breathing [10]. We had no evidence of respiratory complications. Due to the lack of cooperation in the pediatric population, there is a need for higher levels of sedation to approach the airway. Compared with the adult population, where topical anesthesia with or without mild sedation is the gold standard strategy to manage the difficult airway [11], in the pediatric population, that is impossible. Awake fiberoptic intubations are not suitable for pediatric patients. Other drugs used for sedation, like propofol, midazolam, and opioids, have a higher risk of airway patency loss and apnea [2].

## Conclusions

Adequate airway evaluation and planning are essential to minimizing complications when dealing with a pediatric difficult airway. The level of sedation and analgesia provided, hemodynamic stability, and maintenance of the respiratory drive associated with the combination of ketamine and dexmedetomidine make this strategy a promising one for the management of a pediatric difficult airway. Even for an uncooperative adult patient, this may be a valuable strategy. More studies are needed to prove the efficacy and safety of this strategy in the pediatric population.
